# Factors Associated with Postnatal Depression among Mothers Attending at Bharatpur Hospital, Chitwan

**DOI:** 10.1155/2020/9127672

**Published:** 2020-09-22

**Authors:** Mariya Chalise, Isha Karmacharya, Maheshor Kaphle, Ayurma Wagle, Natasha Chand, Laxmi Adhikari

**Affiliations:** Central Institute of Science and Technology, Pokhara University, Kathmandu, Nepal

## Abstract

Postnatal depression is linked with adverse outcomes for mothers, offspring, and her entire family, which stands as a significant public health problem and is often taken as a neglected issue of maternal and child health in the developing world. Postnatal depression is often falsely interpreted as common consequences related to the recent delivery. The main objective of this study is to find out the status of postnatal depression and the factors associated with it among the postnatal mothers attending at Bharatpur Hospital. *Methodology*. A total of 242 postnatal women were included in a hospital-based cross-sectional descriptive study. A systematic random sampling technique was done to get the sampling interval. Face to face interview technique was used for data collection, and depressive symptoms were measured by the Edinburgh Postnatal Depression Scale. Data was entered in Epi-Data and imported to SPSS for analysis. The data were summarized in terms of frequency (percentage), mean (SD), or median (IQR) as per necessity for descriptive analysis. The chi-square test and binary logistic regression were performed to find out the association between the covariates and depression status, assuming significance at *p* value <0.05. *Results*. The study revealed that the prevalence of postnatal depression was 16.9% by EPDS at cutoff point ≥12. It was found that postnatal depression was associated with current age, smoking, pressure to conceive a child, intent of pregnancy, and delivery-related complications. *Conclusion*. Postnatal depression within six months of delivery was found among nearly one-fifth of women, where 13.6% also had suicidal thoughts. More than half of the postnatal women had an early marriage. It is recommended that mothers with high risk should be routinely screened for postnatal depression followed by necessary interventions as well as safe motherhood counseling.

## 1. Introduction

Mental health problems are the crucial public health issues for women of reproductive age in both high- and low middle-income countries [1]. Childbirth signifies for women a time of great vulnerability to develop poor mental health, with postnatal mood disorders, which is the most common form of maternal morbidity following delivery. The prevalence of postnatal depression is especially high in developing countries, where psychological issues are mostly ignored [2]. There is a lack of proper information exchange between the health care providers and mothers regarding the pregnancy and postpartum period mostly among mothers having physical disabilities [3]. The prevalence of PPD reportedly ranges from 0.5% to 60% globally and from 3.5% to 63.3% in Asian countries, in which Pakistan and Malaysia had the highest and lowest rates, respectively [4]. Various studies conducted in Nepal show that the prevalence of PND ranges from 4.9% to 30% [1]. Studies conducted at different times in Maternity Hospital, Dhulikhel Hospital, and Teaching Hospital reported the prevalence of PPD among women who delivered to be 30%, 29%, and 22.2%, respectively [1, 5, 6]. PPD has been linked with catastrophic outcomes, such as maternal suicide and infanticide [7]. The main aim of this research is to study factors associated with postnatal depression among postnatal mothers attending at Bharatpur Hospital, Chitwan.

## 2. Methods

### 2.1. Study Setting and Population

A hospital-based cross-sectional study was conducted from August to September 2019 among postnatal mothers attending Bharatpur Hospital, Chitwan District, Nepal, which is the second-largest government hospital, providing a range of quality healthcare services since seven decades with a large number of patient flow, even from the neighboring districts [7]. Mothers who gave birth in the last six months were included in the study, excluding those who had a preregistered and preexisting history of mental illness and the inability of giving consent.

### 2.2. Sample Size and Technique

The sample size was 242, calculated using data analyst software, at a 95% confidence interval (*z* = 1.96) with allowable error to be 5% and prevalence of depression 22.2% [5]. Bharatpur Hospital was selected purposively, and then, to determine the sample, systematic random sampling was done. The deliveries in the hospital as of FY 2070/71, annual report were 10,292 [8], which estimated 28.58≈29 deliveries per day. Since the allocated time duration for data collection was for 12 days, thus the assumed deliveries in 12 days were 343. A starting point was determined from the list of patients provided in the maternity ward, and then by using sampling interval formula (interval (*i*) = *N*/*n*), participants were selected after every two intervals (343/242).

### 2.3. Study Tool and Variables

A structured interview schedule with close-ended questions was adopted with the author's permission, which was then prepared after an intensive literature review and experts' consultation [9]. All the questions were translated into the Nepali language and back-translated into the English language to maintain translation validity. The questionnaire translated into the Nepali language was pretested among 10% of the total sample, i.e., 24 (*n* = 242) postnatal women of Bhaktapur Hospital, and the same were excluded from the main study. After pretesting, necessary modifications were done, such as stillbirth was excluded from the study.

### 2.4. Dependent Variable

The depressive symptoms for postnatal depression were screened with the Edinburgh Postnatal Depression Scale (EPDS) consisting of 10 questions [10]. Questions 1, 2, and 4 are scored 0, 1, 2, or 3, whereas questions 3, 5–10 are reversed scored. The top box was scored 0, and the bottom box was scored 3 in the former case and vice versa [10, 11]. Out of a total score of 30, the cutoff point for the screening of PPD was found to be 12/13 in the Nepalese context, using 12 as a score for feasibility [12].

### 2.5. Correlates of Postnatal Depression

*Sociodemographic variables* included current age (categorized into less than 25 years and more than 25 years), ethnicity (categorized into privileged and underprivileged), education (categorized into less than secondary and higher than secondary), occupation (categorized into a housewife and working outside the house), and smoking practice of the participants. *Family-related variables* included economic status (categorized into rich and middle class/poor), family type (categorized into nuclear and joint/extended), and family support. *Obstetric-related variables* included parity, intent of pregnancy, ANC service utilization, pregnancy-related complications, delivery-related complications, and mode of feeding (categorized into normal feeding and formula/mixed feeding).

### 2.6. Data Collection and Statistical Analysis

With ethical approval from Nepal Health Research Council, informed consent was obtained from the participants after clarifying the study objectives and ensuring the maintenance of confidentiality, voluntary participation, and right to withdraw at any point in data collection. Face to face interview was conducted among eligible participants.

All the data were entered in Epi-Data version 3.1 and analyzed via SPSS version 20. Descriptive frequency, percentage, mean, and standard deviation were calculated. The chi-square test and binary logistic regression were performed to find out the association between the covariates and depression status, assuming significance at *p* value <0.05.

## 3. Results

### 3.1. Prevalence of Depressive Symptoms among Postnatal Mothers

Out of 242 participants, 41 were found to be suffering from postnatal depression by the EPDS cutoff point at ≥12 [12]. Hence, the prevalence rate of PPD was found to be 16.9%. Among the total participants, 13.6% of them ever had suicidal thoughts during the six months postnatal period.

### 3.2. Sociodemographic Characteristics

Out of 242 participants, the mean age of the participants was 23.8 (SD = ±4.4) with minimum and maximum age being 15 and 37 years, respectively. The majority of the respondents belonged from the underprivileged group (65.3%). The majority of the postnatal mothers (61.6%) had completed secondary level education while only 38.4% of postnatal mothers had education status higher than the secondary level. More than half of the respondents were housewives (78.1%). Among the total respondents, 4.5% of them were smokers while 95.5% were nonsmokers.

### 3.3. Factors Associated with Postnatal Depression

The study showed the current age of postnatal mothers (*p* = 0.024) (Cramer's *V* = 0.226) and smoking (*p* = 0.023) (Cramer's *V* = 0.166) to be significantly associated with postnatal depression with a small effect size in sociodemographic variables. Among family-related variables, the pressure to conceive a child was associated with postnatal depression, i.e., (*p* = 0.021) with a slight (small) association (Cramer's *V* = 0.902). In obstetrics-related variables, the intent of pregnancy (*p* = 0.016) (Cramer's *V* = 0.151) and delivery-related problems (*p* = 0.022) (Cramer's *V* = 0.148) were associated with postnatal depression. Both of the variables had a slight association (Table 1).

In this study, participants who were more than 25 years old were 2.56 times (aOR, 95% CI, 1.06-6.18) more likely to have PPD than the participants who were less than 25 years old. The postnatal mothers who smoked were 5.02 times (aOR, 95% CI, 1.17–21.51) more likely to get depressed than mothers who did not smoke. Similarly, if there were any pressure to conceive a child, the odds that such a female gets depressed is 2.43 times (aOR, 95% CI, 0.87–6.79) higher than a female without pressure to conceive a child. Furthermore, postnatal mothers who had unintended pregnancy (aOR = 2.62, 95% CI, 1.10–6.25) and delivery-related problems (aOR = 2.43, 95% CI, 1.10–5.36) had higher odds of getting depressed (Table 2).

## 4. Discussions

### 4.1. Prevalence of the Postnatal Depression

The finding of the study revealed that the overall prevalence of postnatal depression among mothers attending Bharatpur Hospital was 16.9% which was slightly higher than the study done in Dhanusha District where the prevalence was 15.2% and in another study conducted among Rajbansi women having prevalence 12.27% [8] in Nepal. But in a recent study conducted in Dhulikhel Hospital and the immunization clinic of Maternity Hospital in Kathmandu showed the overall prevalence of depressive symptoms to be 29% and 30%, respectively [1, 5]. Prevalence rates of postnatal depression have also been found at a high rate in developing countries including India and Pakistan ranging from 11 to 49% [1]. The variation in the prevalence of postnatal depression could also be linked to cross-cultural differences [9], how items in EPDS is understood and interpreted by women, the cutoff value of EPDS, sample size, assessment time and method[10], limited availability of mental health specialists, and scarcity of available mental health resources [11].

### 4.2. Association between Postnatal Depression and Sociodemographic, Family, and Obstetrics Variables

In this study, sociodemographic factors such as education, religion, occupation, and ethnicity showed no significant association which was similar to the study conducted in Dhulikhel Hospital in Nepal [5]. However, older female participants were found to be associated with postnatal depression which was similar to the study conducted among Pakistani women [12]. Mothers' education was not associated with postnatal depression in this study. On the contrary, a study conducted among Rajbangsi women in Nepal revealed the association of education with postnatal depression [13]. Mothers' occupation was not associated with postnatal depression in this study which was consistent with a study done in North Indian women in India [14]. This can be attributed to the husband being the primary care provider of the family [15]. Smoking was found to be associated with postnatal depression from sociodemographic variables. Likewise, a study conducted in the Lalitpur district in Nepal also showed a significant association of smoking with PND [16]. The reason for this association might be mothers adopting smoking habit as a form of stress reliever and coping mechanism [17].

Economic condition was significantly associated with postnatal depression among 375 Rajbansi women in Nepal [13] while this study showed no association of economic status with postnatal depression. This might be attributed to the majority of postnatal mothers in this study belonging to the fifth quintile of the IWI category (i.e., rich). Family type was not associated with postnatal depression in this study. However, a study conducted in a tertiary hospital in Delhi, India, showed a significant association of family type with postnatal depression [18]. Various studies have shown that a lack of support from the family increases the risk of postnatal depression [11, 19, 20]. In contrast, the findings of the study revealed that there was no association of postnatal depression with family support which was consistent with the findings from the study conducted in Bangladesh [21]. This study showed a significant association between pressure to conceive a child and postnatal depression which falls as a subcategory under family support.

This study identifies no significant association between parity and occurrence of postnatal depression while parity was associated with PND in a study done in Lalitpur [16]. This study shows that mothers with unintended pregnancy were more likely to develop postnatal depression which was consistent with a study conducted in Dhaka, Bangladesh [21], and Qatar [22]. Unwanted pregnancy might trigger stressful and difficult experiences which can induce stressful motherhood including depressive symptoms [20]. Delivery-related problems and postnatal depression were significantly associated in this study. Consistent with this finding, few other studies showed that maternal- or delivery-related difficulties were linked with postnatal depression [23, 24]. A study done in Dhulikhel Hospital in Nepal showed factors found to be significantly associated with increased risk of depression were pregnancy problems [5] while pregnancy-related difficulties were not found to be associated with PND in this study. A study conducted in Pakistan revealed that short breastfeeding duration is associated with a higher prevalence of depression among mothers [25]. A similar result was obtained from a study conducted among Malaysian mothers [26]. However, this study reveals no association of mode of feeding with postnatal depression. This can be attributed to the majority of mothers following exclusive breastfeeding in this study. The studies have shown that formula feeding or a mixture of both does not facilitate to mother-infant attachment, whereas the risk of postpartum depression can more likely be reduced through exclusive breastfeeding [27].

## 5. Conclusion

This study concluded that nearly one-fifth of postnatal women were suffering from postnatal depression within six months postnatal period, with 13.6% of them have ever had suicidal thoughts. Various factors such as the current age of mothers, smoking, pressure to conceive a child, delivery-related problems, and intent of pregnancy were found to be significantly associated with the development of postnatal depression. Postnatal depression has become a significant public health problem affecting pregnant and postnatal mothers worldwide. In Nepal, limited studies have been conducted in this aspect of maternal health. This study recognizes the importance and needs for postnatal depression screening which must be incorporated as a part of safe motherhood counseling. This research will also act as a baseline for conducting further research in similar settings and aspects.

## Figures and Tables

**Table 1:** Association between sociodemographic, family-related and obstetrics-related characteristics, and postnatal depression (*n* = 242).

Characteristics	Total *n* (%)	Postnatal depression		*p* value
		Absent (201, 83.1%) *n* (%)	Present (41, 16.9%) *n* (%)	
Socio-demographic characteristics				
Current age				*0.024*
<25 years	150 (62.0)	131 (87.3)	19 (12.7)	
≥25 years	92(38.0)	70 (76.1)	22 (23.9)	
Mean = 23.8 ± 4.4 years; Min^m^ = 15, Max^m^ = 37				
Ethnicity				0.524
Privileged	84 (34.7)	68 (81.0)	16 (19.0)	
Underprivileged	158 (65.3)	133 (84.2)	25 (15.8)	
Educational status				0.790
≤Secondary education	149 (61.6)	123 (82.6)	26 (17.4)	
>Secondary education	93 (38.4)	78 (83.9)	15 (16.1)	
Occupation				0.402
Housewife	189 (78.1)	159 (84.1)	30 (15.9)	
Working outside house	53 (21.9)	42 (79.2)	11 (20.8)	
Smoking				*0.023^a^*
No	231 (95.5)	195 (84.4)	36 (15.6)	
Yes	11 (4.5)	6 (54.5)	5 (45.5)	
Family-related characteristics				
Family type				0.132
Nuclear	55 (22.7)	42 (76.4)	13 (23.6)	
Joint/extended	187 (77.3)	159 (85.0)	28 (15.0)	
Economic status				0.410
Rich	104 (43.0)	84 (80.8)	20 (19.2)	
Middle class/poor	138 (57.0)	117 (84.8)	21 (15.2)	
Family support				0.126^a^
Yes	230 (95.0)	8 (66.7)	4 (33.3)	
No	12 (5.0)	193 (83.9)	37 (16.1)	
Pressure to conceive a child				*0.016* ^∗^
No	215 (88.8)	183 (85.1)	32 (14.9)	
Yes	27 (11.2)	18 (66.7)	9 (33.3)	
Obstetrics-related characteristics				
ANC service utilization				0.425
0 or <4 visits	32 (13.2)	25 (78.1)	7 (21.9)	
≥4 visits	210 (86.8)	176 (83.8)	34 (16.2)	
Intent of pregnancy				*0.016* ^∗^
Intended	188 (77.7)	162 (86.2)	26 (13.8)	
Unintended	54 (22.3)	39(72.2)	15 (27.8)	
Pregnancy-related complications				0.051
No	202 (83.5)	172 (85.1)	30 (14.9)	
Yes	40 (16.5)	29 (72.5)	11 (27.5)	
Parity				0.837
Prim parity	151 (62.4)	126 (83.4)	25 (16.6)	
Multiparty	91 (37.6)	75 (82.4)	16 (17.6)	
Delivery-related complications				*0.022* ^∗^
No	145 (59.9)	127 (87.6)	18 (12.4)	
Yes	97 (40.1)	74 (76.3)	23 (23.7)	
Mode of feeding				0.450
Exclusive feeding	111 (45.9)	90 (81.1)	21 (18.9)	
Mixed/formula	131 (54.1)	111 (84.7)	20 (15.3)	

^a^ *p* value from Fisher's exact test; all others are from the chi-square test. ^∗^*p* value significant at <0.05.

**Table 2:** Factors associated with postnatal depression (*n* = 242).

Characteristics	Postnatal depression	
	Unadjusted	Adjusted
	OR (95% CI)	OR (95% CI)
Participant's characteristics		
Current age		
<25 years	Ref.	Ref.
≥25 years	*2.17 (1.10–4.27)* ^∗^	*2.56 (1.06-6.18)* ^∗^
Ethnicity		
Privileged	Ref.	Ref.
Underprivileged	0.80 (0.40–1.60)	0.77 (0.29–2.02)
Educational status		
>Secondary education	Ref.	Ref.
≤Secondary education	1.10 (0.55–2.20)	0.95 (0.37–2.45)
Occupation		
Housewife	Ref.	Ref.
Working outside house	1.39 (0.64–2.99)	1.29 (0.53–3.11)
Smoking		
No	Ref.	Ref.
Yes	*4.51 (1.31–15.58)* ^∗^	*5.02 (1.17–21.51)* ^∗^
Family-related characteristics		
Family type		
Joint/extended	Ref.	Ref.
Nuclear	1.76 (0.84–3.69)	1.72 (0.72–4.13)
Economic status		
Rich	Ref.	Ref.
Middle class/poor	0.75 (0.38–1.48)	0.78 (0.33–1.87)
Family support		
Yes	Ref.	Ref.
No	2.61 (0.75–9.11)	3.31 (0.77–14.23)
Pressure to conceive a child		
No	Ref.	Ref.
Yes	*2.86 (1.18–6.92)* ^∗^	*2.43 (0.87–6.79)* ^∗^
Obstetrics-related characteristics		
ANC service utilization		
≥4 visits	Ref.	Ref.
0 or<4 visits	1.45 (0.58–3.62)	1.22 (0.43–3.48)
Intent of pregnancy		
Intended	Ref.	Ref.
Unintended	*2.40 (1.16–4.95)* ^∗^	*2.62 (1.10–6.25)* ^∗^
Pregnancy-related complications		
No	Ref.	Ref.
Yes	2.18 (0.98–4.82)	1.59 (0.62–4.09)
Parity		
Prim parity	Ref.	Ref.
Multiparty	1.08 (0.54–2.14)	0.82 (0.31–2.14)
Delivery-related complications		
No	Ref.	Ref.
Yes	*2.19 (1.11–4.33)* ^∗^	*2.43 (1.10–5.36)* ^∗^
Mode of feeding		
Exclusive feeding	Ref.	Ref.
Mixed/formula	0.77 (0.39–1.51)	0.60 (0.28–1.30)

^∗^ *p* < 0.05. All other statistics are not significant at *p* < 0.05; model adjusted for all covariates.

## Data Availability

The data used to support the findings of this study are available from the corresponding author Laxmi Adhikari upon request through the email address luxmiadhikari67@gmail.com.
